# Evaluation of the Function of ASFV Gene E66L in the Process of Virus Replication and Virulence in Swine

**DOI:** 10.3390/v15020566

**Published:** 2023-02-18

**Authors:** Elizabeth Ramirez-Medina, Elizabeth A. Vuono, Ayushi Rai, Nallely Espinoza, Alyssa Valladares, Edward Spinard, Lauro Velazquez-Salinas, Douglas P. Gladue, Manuel V. Borca

**Affiliations:** 1 Plum Island Animal Disease Center, Agricultural Research Service, United States Department of Agriculture, Greenport, NY 11944, USA; 2 Oak Ridge Institute for Science and Education (ORISE), Oak Ridge, TN 37830, USA

**Keywords:** ASFV, ASF, African swine fever virus, E66L

## Abstract

African swine fever virus (ASFV) is the etiological agent of an economically important disease of swine currently affecting large areas of Africa, Eurasia and the Caribbean. ASFV has a complex structure harboring a large dsDNA genome which encodes for more than 160 proteins. One of the proteins, E66L, has recently been involved in arresting gene transcription in the infected host cell. Here, we investigate the role of E66L in the processes of virus replication in swine macrophages and disease production in domestic swine. A recombinant ASFV was developed (ASFV-G-∆E66L), from the virulent parental Georgia 2010 isolate (ASFV-G), harboring the deletion of the E66L gene as a tool to assess the role of the gene. ASFV-G-∆E66L showed that the E66L gene is non-essential for ASFV replication in primary swine macrophages when compared with the parental highly virulent field isolate ASFV-G. Additionally, domestic pigs infected with ASFV-G-∆E66L developed a clinical disease undistinguishable from that produced by ASFV-G. Therefore, E66L is not involved in virus replication or virulence in domestic pigs.

## 1. Introduction

African swine fever (ASF) is a usually lethal infectious disease of swine currently presenting as a pandemic affecting several countries in central Europe, Asia and the Caribbean area. The economic consequences of this pandemic are devastating, causing a potential worldwide shortage of protein availability [1]. Since commercial vaccines are only available in Vietnam, disease control in most countries is strictly based on culling susceptible animals and strict biosecurity measures to prevent disease spread.

The etiological agent of ASF, the ASF virus (ASFV), possesses a structurally complex virus particle harboring a 180–190 kilobase pairs double-stranded DNA genome which encodes for 150–160 genes [2]. The functions of most of these genes are unknown, as only a few of them have been experimentally characterized, and information is limited to structure predictions [3], or limited viral–host protein interaction screens [4,5,6,7,8,9]. Understanding the role of viral proteins in the process of virus replication and/or virus virulence is critical to developing novel countermeasures for disease control. In fact, ASFV experimental vaccines produced by deleting specific virus genes have been shown to be the most effective in inducing protection against the current circulating strains in Europe and Asia, and all derivatives of the ASFV Georgia 2007 isolate [10,11,12,13,14,15,16,17]. To date, all ASF vaccines were developed by identifying and genetically manipulating virus genes implicated in the process of disease production toward the production of attenuated virus strains [18]. Therefore, understanding the role of individual genes and how their manipulation could be used to develop novel countermeasures is of paramount importance.

Without experimental information about viral gene function, the systematic deleting of specific virus genes by rationally manipulating the virus genome is a particularly efficacious methodological approach to understand the role of a specific gene in critical virus functions such as virus replication and disease production, in some instances a single deletion or double deletion attenuates the virus and offers protection against homologous challenge [18]. However, in some cases a single gene deletion does not display a phenotype, for example, when ASFV genes I8L, X69R, TK, MGF-110-1L or MGF-360-1L [19,20,21,22,23] were deleted, perhaps due to a duplicated function or partial function with another ASFV gene.

The ASFV E66L gene encodes for a virus protein that has been implicated in decreasing host gene expression. In particular, the transmembrane domain of E66L was involved in suppressing mRNA translation at the endoplasmic reticulum, modulating the PKR/eIF2a pathway producing host translation downregulation [24]. Here, we present results evaluating the involvement of E66L during ASFV replication in swine macrophage cultures and during experimental infection in domestic pigs.

## 2. Materials and Methods

### 2.1. Viruses and Cells

Cultures of primary swine macrophage were developed from peripheral blood as was previously described [25]. Blood donors were hosted in biosafety level 3 conditions at the Plum Island Animal Disease Center (PIADC) (Orient, NY 11957, USA) animal facility and handled under protocol approved by the Institutional Animal Care and Use Committee (IACUC) (205.06-20-R_090716). Peripheral blood mononuclear cells were enriched using a Ficoll-Paque (Pharmacia, Piscataway, NJ, USA) density gradient followed by incubation for 24 h at 37 °C under 5% CO2 in Primaria flasks. The adherent cell fractions were then detached from and reseeded into Primaria T25, 6- or 96-well dishes at a density of 5 × 10^6^ cells per mL for further use. ASFV Georgia (ASFV-G) was a field isolate kindly provided by Dr. Nino Vepkhvadze from the Laboratory of the Ministry of Agriculture (LMA) in Tbilisi, Republic of Georgia [26].

ASFV-G-∆E66L and parental ASFV-G growth curves were performed in 24-well plates of primary swine macrophage cell cultures at an MOI of 0.01 (based on HAD_50_, 50% hemadsorbing doses, as previously determined in primary swine macrophage cell cultures). After adsorption for 1 h at 37 °C under 5% CO2, the inoculum was removed. Cells were then rinsed twice with PBS and further incubated with macrophage media at 37 °C under 5% CO2. At specific times post-infection (2, 24, 48, 72, and 96 h post infection), cells were frozen at ≤−70 °C and the thawed lysates were used to calculate virus titers by HAD50/mL in 96-well plates cultures of primary swine macrophage. Similarly, viremias were also quantified in swine macrophage cultures in 96-wells plates. Presence of the virus was assessed by hemadsorption (HA) and virus titers were calculated as previously described [27].

### 2.2. Construction of the E66L Deletion Mutant ASFV

An ASFV harboring the deletion of the E66L gene (ASFV-G-∆E66L) was developed by homologous recombination between the genome of the highly virulent ASFV-G strain and a recombination vector (p72mCherryΔE66L) following a protocol previously described [10]. p72mCherryΔE66L harbors the flanking genomic regions of the E66L gene with the left flanking region situated between genomic positions 169484 and 170484 and the right flanking region situated between genomic positions 170604 and 171604. p72mCherryΔE66L also contains a reporter gene cassette with the mCherry fluorescent protein (mCherry) gene under the control of the ASFV p72 late gene promoter [28]. p72mCherryΔE66L vector was obtained by DNA synthesis (Epoch Life Sciences, Sugar Land, TX, USA). As designed, the construct should create a 119bp nucleotide deletion leaving only the first 35nt of the gene to not disturb the termination sequence in gene I267L deleting the remaining 119bp of the E66l gene sequence. The recombinant ASFV-G-∆E66L was purified by 14 successive limiting dilution steps being positively selected based on the presence of fluorescence. ASFV-G-∆E66L genome was full-length sequenced using next-generation sequencing (NGS) performed as previously described [28] using an Illumina NextSeq500 sequencer. Sequence analysis was performed using CLC Genomics Workbench software version 20 (QIAGEN, Hilden, Germany).

### 2.3. Animal Experiments

The level of virulence of the recombinant ASFV-G-∆E66L was assessed by experimentally inoculating 35–40 kg commercial breed swine. Five pigs were intramuscularly (IM) inoculated with 10^2^ HAD_50_ of ASFV-G-∆E66L and the effect was compared with a similar group of pigs inoculated with 10^2^ HAD_50_ of the parental ASFV-G. Presence of clinical signs of ASF (anorexia, depression, fever, purple skin discoloration, staggering gait, diarrhea, and cough) as well as changes in body temperature were recorded daily throughout the experiment. Animal experiments were performed in biosafety level 3 conditions at the PIADC animal facility, under protocol approved by the IACUC (225.01-16-R_090716).

### 2.4. Statistical Analysis

Differences between ASFV-G and ASFV-G-∆E66L including growth kinetics, body temperature and levels of viremia in the inoculated animals were conducted by the unpaired *t* test (*p*-value 0.05) assuming individual variances for each row, being multiple comparisons conducted by the false rate discovery rate approach. To minimize the presence of false positives (*q*-value 0.05) the two-stage set-up (Benjamini, Krieger, and Yekutieli) method was applied. On the other hand, survival curve comparisons between groups of animals inoculated with ASFV-G and ASFV-G-∆E66L were conducted using the log-rank (Mantel–Cox) test (*p*-value 0.05). All statistical analyses were conducted in the software GraphPad Prism 9.5.0. 

## 3. Results and Discussion

### 3.1. Evolutionary Dynamics of E66L among ASFV Isolates in Field

To assess the genetic variability of the E66L gene in nature, a blast analysis was performed using this gene from the ASFV isolate Georgia 2007 (GenBank access NC_044959.2) as a query. As a result, a total of eight representative ASFV isolates associated with diverse genotypes (I, II, III, IV, V and VII) were identified for further assessment.

**Figure 1:**
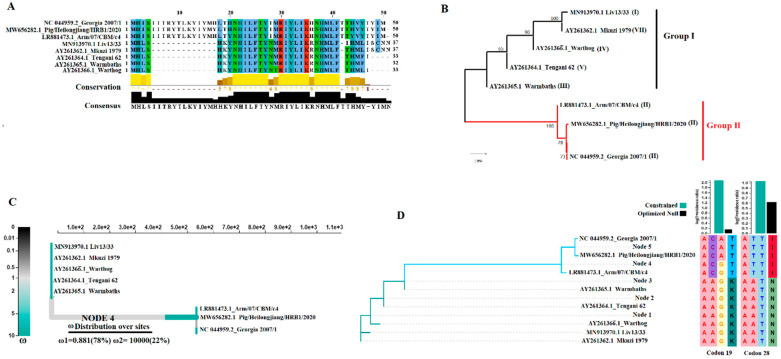
Evolutionary dynamics of E66L gene of ASFV. (**A**) Amino acid alignment showing the diversity of E66L protein among a group of representative ASFV isolates. Conservation plot scores reflect the nature of the change in specific sites. Increased scores reflect substitutions between residues with similar biological properties. Transmembrane domain (TMD) boundaries are limited by a red square. Analysis was conducted in the software Jalview version 2.11.1.7. (**B**) Phylogenetic analysis conducted by neighbor joining method using the full-length sequence of E66L gene supporting the existence of two phylogenetic groups. Numbers in the parenthesis indicate the genotype of different strains based on p72 classification. (**C**) aBRISEL analysis. Phylogenetic tree showing the w (dN/dS) rates at different branches associated with representative isolates included in this study. Evidence of positive selection was found on node 4, where two different classes of w (w1 and w2) were predicted. *w* values < or > than 1 are associated with negative or positive selection, respectively. Percentages in the parenthesis represent the proportion of codon sites associated with each class. (**D**) BUSTED analysis. Prediction of the potential codon site where evidence for the rejection of the null hypothesis (absence of positive selection) was observed.

Interestingly, blast analysis conducted on other ASFV isolates (available full-length sequences), including Malawi Lil-20/1 and SPEC-57 (genotype VIII), Ken05/Tk1, R7, R8, R35, R25, N10 (genotype IX), Kenya 1950 and Ken06.Bus (genotype X), RSA-W1-1999 (IV), RSA-2-2004 (XX) and RSA2/2008 (XXII), produced negative results, indicating the absence of the E66L gene in multiple isolates of ASFV. Conversely, the E66L sequence present in the isolate Pretoriuskop/96/4 associated with the genotype XX, appeared like the isolate Tengani 62 associated with genotype V. It is interesting, considering the absence of E66L gene in other sequences from genotype XX, suggesting that introduction of E66L gene in different isolates may be the resultant of a recombination process. Future analyses are needed to obtain more insights into this possibility. Pairwise analysis, using the p-distance model and the bootstrap method to give statistical confidence to the inferences (*p* < 0.05), revealed an identity between 26.03 and 99.06% (~70.89%), and 65.71 and 97.22% (~81.84%) at nucleotide and amino acid levels, respectively. The low levels of identity among ASFV isolates are shown in the amino acid alignment presented in Figure 1A. In this context, the results indicated the existence of E66L phenotypes of variable sizes, ranging from 32 to 50 amino acids (Figure 1A). An interesting feature among the isolates was the presence of a 13-amino acid insertion between residues at positions 5 and 17. This insertion is present only in the representative ASFV isolates of the Eurasian lineage (Georgia 2007/1 and Pig/Heilongjiang/HRB1/2020) and the genotype I (Arm/07/CBM/c4) but is absent from the rest of the isolates. Interestingly, this insertion spans the previously predicted transmembrane domain (TMD) from residues 13 to 34 [29], making this domain shorter for some of the isolates (Figure 1A). The TMD was characterized by those authors as critical for shutting down host gene translation.

Considering the differences between isolates, a phylogenetic analysis was performed using the neighbor joining method with maximum likelihood as model and 1000 bootstrap replicates [30]. Consistent with the low levels of identity among isolates, the phylogenetic analysis inferred the existence of at least two phylogenetic groups, representing the different forms of E66L gene in nature (Figure 1B). In this sense, pairwise distance analysis conducted between groups revealed an overall identity between groups of 81.70%. To detect independent mutations putative between both groups, a chi-square analysis of independence using the software metadata-driven comparative analysis tool was conducted [31]. The results identified 11 significant putative mutations between groups (*p*-value 0.046): three of them were predicted at synonymous sites in the alignment at positions 4, 99 and 135, while the rest at positions 7, 52, 53, 54, 56, 83, 107 and 133 were located at nonsynonymous sites at multiple codons. From these positions, sites 52, 53, 54 (amino acid 18), 56 (amino acid 19) and 83 (amino acid 28), were found to impact the previously predicted TMD, indicating potential phenotypic difference between both groups in this domain.

Interestingly, not only the presence of the insertion at this protein, but also the identification of putative sites between the different groups, may have potential implications in the molecular epidemiology of ASFV. These findings support potential discrimination between viruses associated with genotype II and the rest of the ASFV isolates associated with distinct genetic groups. 

Finally, to gain more insight into the evolution of the E66L gene in nature, we conducted a systematic evolutionary analysis as previously published for SARS-CoV-2 [32], and ASFV [33,34,35,36]. Analysis by the single-likelihood ancestor counting (SLAC) algorithm [33] revealed dN (synonymous substitution rate)/dS (nonsynonymous substitution rate) ratio = 1.21. This fact was consistent with the increased number of putative nonsynonymous mutations predicted between the two genetic groups. Interestingly, this situation contrasted with our previous predictions at, A859L [34], A151R [35], A104R [36], E165R [37], EP296R [38], and H108R [39], where variable dN/dS rates <1 were predicted; therefore, indicating that positive selection is the dominant force shaping the evolution of E66L gene.

Based on the predicted genetic diversity of E66L in nature and the high dN/dS values, we hypothesized that the divergence between phylogenetic groups one and two might have been driven by natural selection. To test this hypothesis, the evolutionary algorithm aBRISEL (adaptive branch-site random effects likelihood) was used [40]. Interestingly, the results by aBRISEL analysis supported the hypothesis, suggesting that the divergence between both groups was mediated by positive selection. A significant result (LRT = 10.48, *p*-value = 0.020) was obtained in the predicted ancestral node 4, the long branch associated with divergence between both groups. At this branch, 22% of the codons were predicted with high w (dN/dS) values (Figure 1C). These results were confirmed by the algorithm BUSTED (branch-site unrestricted statistical test for episodic diversification) [41]. In this sense, BUSTED was used to evaluate different codon sites at multiple branches on the tree under the constrained and the optimized null models (both disallowing positive section). As result, we identified, at codons 19 and 28, evidence about the rejection of the constrained and the optimized null models, indicating the potential relevance of these sites in promoting the divergence between both groups. Interestingly, these two sites are located at the previously predicted TMD [29], suggesting that mutations on residues at positions 19 and 28 may represent a framework for future research studies on the function of the E66L protein. In light of these results, we may warn about the existence of potential functional differences in the E66L protein between these two groups.

Additionally, no evidence of recombination was predicted on E66L after evaluation by GARD (genetic algorithm for recombination detection) [42], suggesting that recombination is not playing a role in the evolution of E66L protein.

### 3.2. Development of a Recombinant ASFV-G-ΔE66L Deletion Mutant

**Figure 2:**
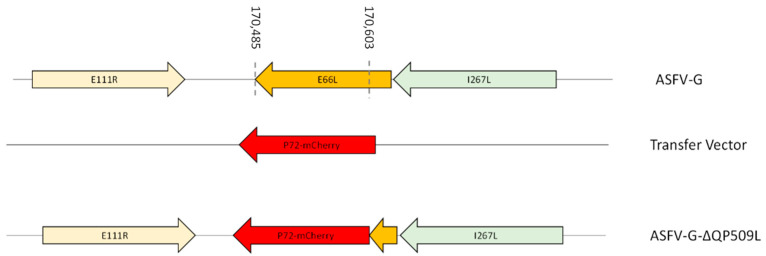
Schematic for the development of ASFV-G-∆E66L. The transfer vector contains the p72 promoter and an mCherry cassette; the flanking left and right arms are indicated. They were designed to have flanking ends to both sides of the deletion/insertion cassette. The nucleotide positions of the ASFV-G genome are indicated. The resulting ASFV-G-∆E66L virus with the cassette inserted is shown on the bottom.

To understand the influence of the E66L gene function in the processes of ASFV replication in macrophage cell cultures and disease production in swine, a recombinant ASFV-G virus harboring the deletion of the E66L gene (ASFV-G-∆E66L) was developed. The recombinant ASFV-G-∆E66L presents the substitution of the E66L gene with the p72mCherry cassette produced by homologous recombination [28]. A genomic area covering 119-bp (situated between nucleotide positions 170485 and 170603) was deleted from the ASFV-G genome and further substituted with the p72mCherry cassette (see Material and Methods) (Figure 2). The initial stock of ASFV-G-∆E66L was purified after successive limiting dilution steps in primary swine macrophage cell cultures. The final ASFV-G-∆E66L stock was developed by amplifying the virus obtained in the last purification round, also using primary swine macrophage cell cultures.

The precision of the genomic changes introduced during the development of ASFV-G-∆E66L, as well as the integrity of the full genome of the virus, was assessed by NGS using an Illumina NextSeq 500. The analysis of the information corroborates the expected deletion of 119 nucleotides, consistent with the designed genomic modifications, as well as the insertion of the p72-mCherry cassette sequence. No other undesired genetic differences were found between ASFV-G-∆E66L and ASFV-G. Genomes indicating no unwanted genetic changes were introduced during the process of development and purification of ASFV-G-∆E66L. Additionally, NGS data confirmed the absence of any ASFV-G genome, eliminating the presence of a potential contamination with parental virus in the stock of ASFV-G-∆E66L.

### 3.3. Replication of ASFV-G-ΔE66L in Primary Swine Macrophages

**Figure 3:**
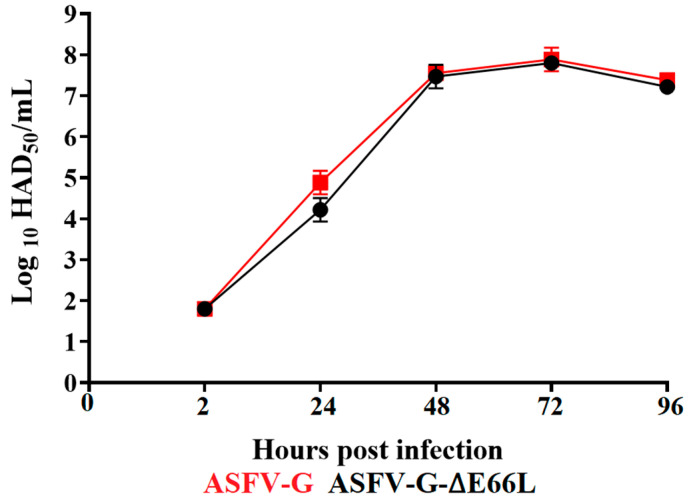
In vitro growth kinetics in primary swine macrophage cell cultures for ASFV-G-∆E66L and parental ASFV-G (MOI = 0.01). Samples were taken from three independent experiments at the indicated time points and titrated. Data represent means and standard deviations of the virus titers. Sensitivity using this methodology for detecting virus is ≥log10 1.8 HAD_50_/mL. No significant differences in viral yields between viruses were observed at any time point tested using the two-stage set-up (Benjamini, Krieger, and Yekutieli) method.

To understand the possible role of the E66L gene during the process of virus replication, the growth ability of ASFV-G-∆E66L was evaluated and compared to that of the parental ASFV-G performing a multistep growth curve of primary swine macrophage cultures. Macrophage cultures were infected (MOI of 0.01) with either ASFV-G-∆E66L or ASFV-G, and virus yields were quantified at 2, 24, 48, 72, and 96 h post-infection (pi). Results showed that the recombinant ASFV-G-∆E66L presented a growth kinetic that was practically indistinguishable from that of the parental ASFV-G. No significant differences in virus titer were detected at any of the time points assessed (Figure 3). Therefore, the deletion of the E66L gene from the genome of the highly virulent isolate ASFV-G does not significantly alter the capacity of the virus to replicate in swine macrophages, ASFV’s natural target cell during the infection in pigs.

### 3.4. Assessment of ASFV E66L in the Process of Virulence in Domestic Swine

**Figure 4:**
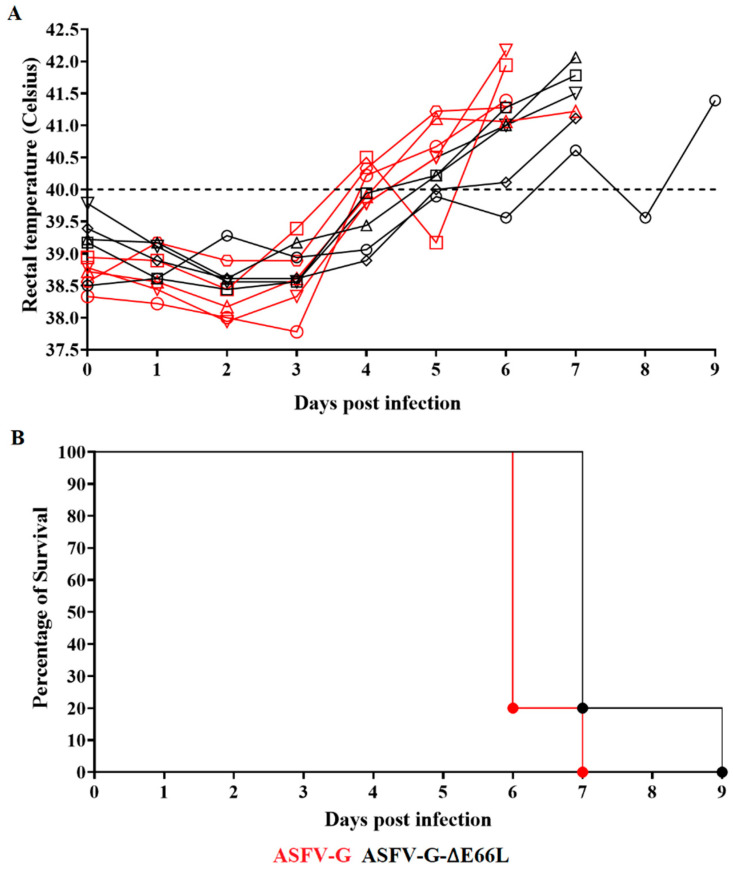
Evolution of body temperature (**A**) and lethality (**B**) in animals (5 animals/group) IM infected with 10^2^ HAD_50_ of either ASFV-G-∆E66L or parental ASFV-G. No statistical differences were found in body temperatures between pigs in both groups when evaluated by the two-stage set-up (Benjamini, Krieger, and Yekutieli) method. Conversely, significant differences (*p*-value = 0.0201) in the survival course between groups of pigs were found using the log-rank test (Mantel–Cox test).

To understand the potential consequences of the deletion of the E66L gene on the process of disease production in domestic pigs, a group of five pigs (weighing 35–40 kg) was IM inoculated with 10^2^ HAD_50_ per animal while an additional group was inoculated under similar conditions with the virulent parental ASFV-G. As expected, all animals inoculated with the parental virulent ASFV-G showed an increase in body temperature (>40 °C) by day 4–5 pi followed by the fast worsening of clinical signs associated with ASF (Figure 4). All animals needed to be euthanized *in extremis* between days 6–7 pi due to the severity of the clinical signs.

The animals inoculated with ASFV-G-∆E66L also developed an acute clinical form of the disease characterized by a rise in body temperature by day 5–6 pi and the appearance of ASF-related clinical signs that progressively worsened in the following days, with four animals euthanized on day 7 pi and the last one on day 9 pi. The kinetics of the presentation of clinical signs between the two groups of animals indicated that deletion of the E66L gene from the genome of the highly virulent isolate ASFV-G does not significantly affect virus virulence during experimental infection in domestic pigs.

**Figure 5:**
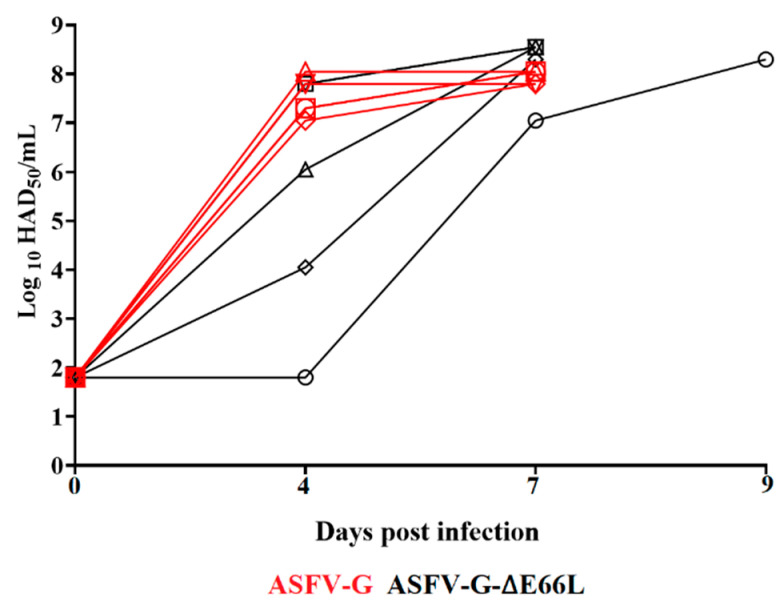
Viremia titers detected in pigs IM inoculated with 10^2^ HAD_50_ of either ASFV-G-∆E66L (filled symbols), or ASFV-G (empty symbols). Each symbol represents the average of animal titers in each of the groups. Sensitivity of virus detection: >log10 1.8 TCID_50_/mL. No significant differences in viremia values between both groups of pigs were found using the two-stage set-up (Benjamini, Krieger, and Yekutieli) method.

The level of systemic replication of either virus in the inoculated animals was evaluated by quantifying viremia titers during the experiment. Animals infected with the virulent ASFV-G showed, as expected, high titers of viremia (ranging from 10^7^ to 10^8^ HAD_50_/mL) by day 4 pi, evolving to even higher titers until the day animals were euthanized. Animals inoculated with ASFV-G-∆E66L showed a wide array of viremia values ranging from undetectable (≤10^1.8^ HAD_50_/mL) to 10^7.8^ HAD_50_/mL by day 4 pi, reaching maximum titers by day 7 pi, when all animals were euthanized (Figure 5). Therefore, although statistical differences were transiently found in the average of viremia titers at 4 dpi at the time of euthanasia, viremia titer values were indistinguishable between animals inoculated with the recombinant ASFV-G-∆E66L or the parental virulent virus.

The results presented here indicate that the deletion of E66L from the genome of highly virulent parental ASFV-G does not produce a drastic effect on the process of virus replication or disease production in domestic pigs. Full length genomic sequences, obtained by NGS, of virus isolated from euthanized animals experimentally inoculated with ASFV-G-∆E66L confirmed that ASFV-G-∆E66L was responsible for the virulent phenotype. Blood samples obtained from three animals confirmed the absence of any significant differences with the full-length genomic nucleotide sequence of the ASFV-G-∆E66L stock eliminating the possibility that disease in these animals may be caused by the presence of the virulent parental virus contaminating the ASFV-G-∆E66L stock.

## 4. Conclusions

In summary, it has been shown here that the E66L gene does not play an essential function for supporting ASFV growth since its deletion did not affect virus replication either in swine macrophage cultures, or during the experimental infection in pigs. In addition, the presence of E66L did not appear to be critical in the process of ASFV virulence in domestic pigs. These results are unexpected, based on previous reports indicating that the protein encoded by E66L is involved in shutting down host-cell protein expression during the virus infection. We previously reported similar results with ASFV proteins where we initially characterized their importance for virus replication in primary cell cultures. In some cases, virus replication in cell cultures is not affected by a single viral gene deletion [33,36]. As in this case, deletion of E66L from the genome of the virulent ASFV-G produced a recombinant virus that did not decrease the ability to replicate in swine macrophage cell cultures or to cause attenuation when experimentally inoculated in pigs. In cases such as these, where a single gene deletion does not have an apparent effect in cell culture or in swine, the possibility of the existence of another ASFV protein that possesses a compensatory or overlapping activity with the E66L function should be considered. Further research will be necessary to clarify this hypothesis, as the genetic functions of ASFV proteins are further elucidated.

## Data Availability

Data is contained within the article.
